# Cumulative knee adduction moment during jogging causes temporary medial meniscus extrusion in healthy volunteers

**DOI:** 10.1007/s10396-023-01288-w

**Published:** 2023-02-17

**Authors:** Yosuke Ishii, Takato Hashizume, Saeko Okamoto, Yoshitaka Iwamoto, Masakazu Ishikawa, Yuko Nakashima, Naofumi Hashiguchi, Kaoru Okada, Kazuya Takagi, Nobuo Adachi, Makoto Takahashi

**Affiliations:** 1 Department of Biomechanics, Graduate School of Biomedical and Health Sciences, Hiroshima University, Hiroshima, Japan; 2 Department of Orthopaedic Surgery, Faculty of Medicine, Kagawa University, Kagawa, Japan; 3 Department of Musculoskeletal Ultrasound in Medicine, Graduate School of Biomedical and Health Sciences, Hiroshima University, Hiroshima, Japan; 4 Department of Orthopedic Surgery, Graduate School of Biomedical and Health Sciences, Hiroshima University, Hiroshima, Japan; 5 Ultrasound Business Operations, Healthcare Business Headquarters, KONICA MINOLTA, INC, Tokyo, Japan

**Keywords:** Medial meniscus extrusion, Ultrasonography, Cumulative load, Jogging, Uphill/downhill loading, Knee adduction moment, Healthy individual

## Abstract

**Purpose:**

The cumulative knee adduction moment (KAM) is a key parameter evaluated for the prevention of overload knee injuries on the medial compartment. Medial meniscus extrusion (MME), typical in hoop dysfunctions, is a measure for the cumulative mechanical stress in individual knees; however, its correlation with cumulative KAM is unknown. The aim of this study was to investigate the effect of temporary overload stress on MME and its correlation with cumulative KAM.

**Methods:**

Thirteen healthy asymptomatic volunteers (13 knees) were recruited for a cohort study (mean age, 23.1 ± 3.3 years; males: *n* = 8). The cumulative KAM was calculated using a three-dimensional motion analysis system, in addition to the number of steps taken while jogging uphill or downhill. MME was evaluated using ultrasound performed in the standing position. The evaluations were performed four times: at baseline (T0), before and after (T1 and T2, respectively) jogging uphill or downhill, and 1 day after (T3) jogging. Additionally, the Δ-value was calculated using the change of meniscus after efforts as the difference in MME between T1 and T2.

**Results:**

The MME in T2 was significantly greater than those in T0 and T1. Conversely, the MME in T3 was significantly lesser than that in T2. No significant difference was found between those in T0 and T1, and T3. ΔMME exhibited a significant positive correlation with the cumulative KAM (*r* = 0.68, *p* = 0.01), but not for peak KAM.

**Conclusion:**

The temporary reaction of MME observed in ultrasound correlates with the cumulative stress of KAM.

## Introduction

A healthy knee maintains the homeostasis of the cartilage through the correct balance between the loading and metabolic stresses. However, homeostasis can become unbalanced due to extreme mechanical stress through participation in sports and other taxing activities [1–3]. The cumulative stress consequently changes the structure of the knee compartment, eventually resulting in knee injuries and osteoarthritis (OA) [4–6]. It therefore becomes necessary to detect abnormal mechanical stress in the early stages to prevent lesions in the knee.

The knee adduction moment (KAM) is a representative joint loading stress and an indicator of the mechanical stress in the medial compartment of the knee [7]. The KAM can be quantified for evaluation of the loading stress in the stance phase of the single gait cycle and consequently predicting potential OA progression [8]. Nevertheless, the KAM does not provide information about personal and environmental factors, and is hence limited by individual adaptation. However, the cumulative KAM, including the number of steps taken during daily activities, is comprehensive information and more distinguished between individuals with and without OA, including the severity of the condition [9]. The cumulative KAM is thus important for understanding the mechanism of the pathological knee. However, whether this indicator can be adopted for the variable structures in individual knees is unknown.

The meniscus functions as a hoop, effectively distributing the load on the knee and absorbing shock [10, 11]; this is directly correlated with the pathological condition of the knee cartilage [12]. Medial meniscus extrusion (MME) is a characteristic feature of hoop dysfunction [11, 13, 14] and an accurate indicator of the early progression of knee OA due to increased mechanical stress [15–17]. The behavior of MME happens under weight-bearing conditions and is gradually greater after repeated mechanical stress from daily activity [18, 19]. In particular, a previous study demonstrated the behavior of MME during walking, and it was correlated with the KAM [20], and showed that it might contribute to greater MME in knee OA. However, even when knee OA is absent, MME was greater as a result of cumulative load during a mountain marathon in a previous study [21]. Therefore, MME may be reflected in the cumulative KAM, indicating the loading stress in individual knees.

This study aimed to investigate the effects of cumulative stress on MME and cartilage in healthy knees and assess the correlation. We also hypothesized that the MME and cartilage are temporarily exacerbated after jogging uphill and downhill, and that there is a correlation with the cumulative KAM.

## Materials and methods

### Participants

Thirteen healthy volunteers were recruited in this cohort study, with a total of 13 knees for assessment (mean age, 23.2 ± 2.9 years; BMI: 22.1 ± 2.8 kg/m^2^; men: *n* = 8). All participants provided informed consent, and the study was approved by our institution’s ethics department (E-2495–1). The patients participated in recreational sports once a week and had no history of knee injuries, trauma, and surgical interventions. No participant had experienced lower extremity injuries within 3 months preceding this study, nor suffered from chronic pain. These factors would negatively impact their ability to jog. Their characteristics are summarized in Table 1.

**Table 1:** Participant characteristics

	Participants
N/knees	13/13
Gender (M: F)	8: 5
Age (years)	23.2 ± 2.9
Height (cm)	168.4 ± 6.5
Weight (kg)	63.1 ± 11.6
BMI (kg/m^2^)	22.1 ± 2.8

The values represent the means ± standard deviation

*BMI* body mass index

### Study protocol and assessment

**Fig. 1:**
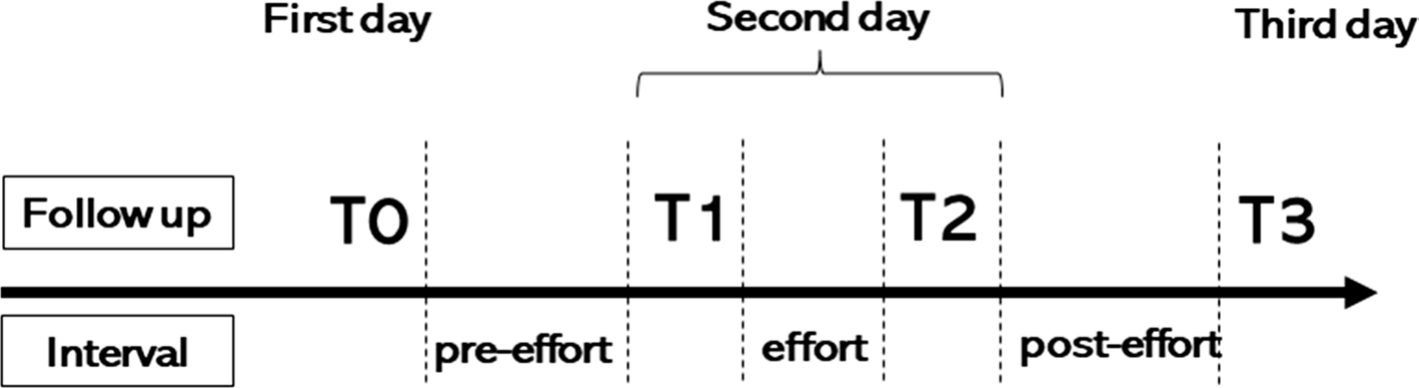
Overview of the study protocol. MME: medial meniscus extrusion, T0: baseline, T1: pre-effort, T2 post-effort, T3: after 1 day of effort, Interval-pre-effort: T0 and T1, Interval-effort: T1 and T2, Interval-post-effort: T2 and T3

This cohort study adhered to the established protocol for 3 days (Fig. 1). Follow-ups were performed at four different time points: at baseline (T0) on the first day, before and after (T1 and T2, respectively) jogging uphill and downhill on the second day, and 1 day after (T3) jogging on the third day. Moreover, the intervals of time were divided between T0 and T1, T1 and T2, and T2 and T3, defined as the following intervals: pre-effort, effort, and post-effort, respectively (Fig. 1).

Motion analysis was performed at T0, and evaluation of MME and cartilage was performed at all follow-ups. The activity data were recorded at all intervals.

### Jogging environment

All participants used the same shoe type (ME-WE432; New Balance, China) in their correct shoe sizes to minimize biases due to differences in shoe properties. On the jogging course, the terrain was flat, uphill, and downhill with an altitude of 70 m. Jogging was done at a comfortable speed, covering a distance of 5 km.

### Activity levels during jogging and daily activities

The activity data at all intervals were obtained through wearable sensors (DynaPort MoveMonitor; McRoberts, The Hague, the Netherlands) and running watches (ForeAthlete 55; Garmin, USA).

The participants’ daily activities were evaluated through tri-acceleration devices (DynaPort MoveMonitor; McRoberts, The Hague, the Netherlands) with a sampling rate of 100 Hz. The participants were instructed to wear the device during the interval between pre-effort and post-effort according to the study protocol, with the exception of aquatic activities. An elastic belt was used to fix the device dorsally, at the level of L5. The participants’ daily activities were quantified in terms of motion, frequency, and duration based on acceleration data. Extreme stress, except when jogging, could also be monitored. A product analysis system (DynaPort MoveMonitor; McRoberts) automatically monitored this information through motion sensing [22].

The running watch was attached to the participant’s wrist and recorded all information, like steps, pitch, and length while jogging. The step data combined with motion analysis data were used to calculate the cumulative loading. The participants returned from the load course to our laboratory when their running watches indicated the distances exceeded 5 km, and their meniscus and cartilage were immediately analyzed.

### Motion analyses

Biomechanical evaluations were performed using a three-dimensional motion analysis system at T0. This system was constructed using 16 cameras (VICON612; Vicon Motion Systems, Oxford, UK) and eight force platforms (AMTI, Watertown, Mass) with sampling frequencies of 100 and 1000 Hz, respectively.

Passive markers were attached to designated locations on their bodies, and a model of the plug-in gait (Vicon^®^ Peak, Vicon Motion Systems) was applied. The device settings were adjusted such that the camera had the minimum tracing error because of the calibration system. Subsequently, the joint axes in spatial cording were determined on the static standing position. Jogging was performed for 10 m at individual comfortable speeds, repeated three times.

The raw data from these trials were filtered using the Butterworth fourth order filter with a cutoff frequency of 6 Hz. The analyzed section was a stance phase, including the heel contact to toe-off. These events were determined using a 10-N threshold of vertical grand reaction on force plates. Finally, the knee angle, moments, and spatiotemporal parameters were obtained using the plug-in software (Vicon^®^ Peak, Vicon Motion Systems). Moreover, the cumulative loading was calculated using the participant’s knee moment and the number of steps taken. This calculation of cumulative loading, based on a previous study that reported high reliability [23], was calculated by multiplying the moment impulse with the number of steps in the index limb. The average of three trials was used for the statistical analyses.

### Evaluation of meniscus extrusion and cartilage

**Fig. 2:**
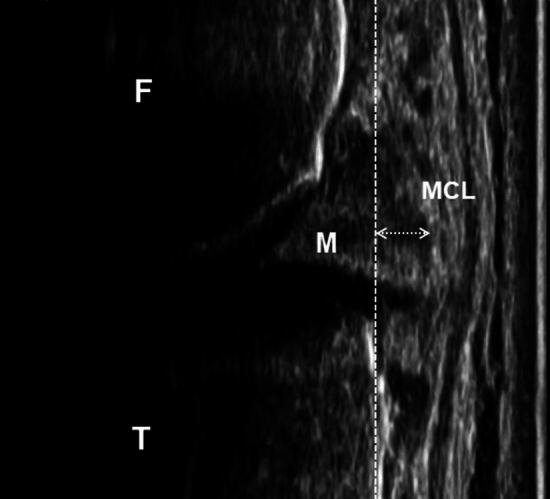
Medial meniscus extrusion. The coronal image shows the medial meniscus in the standing position. M: medial meniscus, MCL: medial collateral ligament, F: femur, T: tibia. The dash line shows the distance of the meniscus extrusion drawn between the outmost edge of the meniscus and the cortex in the tibial plateau

The medial meniscus was evaluated using ultrasound (SNiBLE; Konica Minolta, Japan) with a linear transducer (3–11 MHz; Konica Minolta, Japan). A vertical line was drawn on the participant’s knee, between the top of the medial condyle to the proximal tibia, such that the medial collateral ligament was clearly visible on the image. A longitudinal transducer was placed on the medial joint space on a vertical line with knees at full extension. Images were captured when the bound between the medial collateral ligament and medial meniscus on the screen was most visible (Fig. 2). This method had been previously demonstrated to be highly reliable [24] and was performed in the supine and standing positions.

**Fig. 3:**
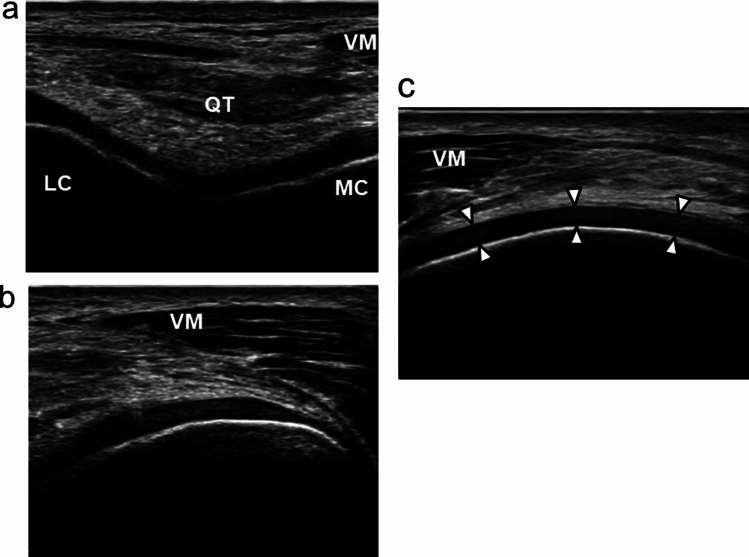
Ultrasound evaluation of cartilage. VM: vastus medialis, QT: quadriceps tendon, LC: lateral condyle, MC: medial condyle. The coronal image of femoral condyles at the level of the upper patella (**a**), the center of the medial condyle in the image (**b**), and cartilage on a longitudinal image (**c**). The arrows at the middle indicate the cartilage thickness

The cartilage was assessed at the thickness on medial femoral condyles in a longitudinal plane, based on a previous study with high validity and reliability, for comparison with magnetic resonance imaging [25]. First, the transducer was transversely placed at the participant’s upper patella at full flexion of the knee in the supine position and adjusted to the medial and lateral condyles line (Fig. 3a). Second, the probe was shifted to the medial side, and the image confirmed that the medial condyle was located at the center (Fig. 3b). The probe was continuously turned from the transverse to longitudinal direction on the knee, and the image was captured in the longitudinal plane (Fig. 3c).

The extent of meniscal extrusion was measured using the distance from the cortical line of the tibial plate to the outermost edge of the meniscus [26] (Fig. 2). The cartilage was identified as the average distance of difference in hyperechoic lines between synovial space–cartilage and cartilage–bone in the interface at three locations: anterior, middle, and posterior (Fig. 3c). Moreover, we represented the post-jogging changes in the meniscus and cartilage as the Δ-values, calculated as the difference in MME and cartilage thickness between time points T1 and T2. These processes were repeated three times, and the average value was adopted for use in the statistical analyses. These measurements were analyzed using the Kinovea software (v0.8.15; Kinovea open-source project, www.kinovea.org).

To calculate the reliability, the MME and cartilage thickness were compared between T0 and T1, and the intra-class correlation coefficient (ICC 1.3) (Y.I) was indicated.

### Statistical analysis

The normality of all datasets was confirmed by Shapiro–Wilk test. For comparing the MME and cartilage thickness during follow-ups, analysis of variance with repeats was performed. Bonferroni corrections were used in the post hoc test if a significant difference was observed in the follow-ups. Moreover, to investigate the correlation between the biomechanical parameters and Δ-values of the meniscus and cartilage, Pearson or Spearman correlation analyses were performed.

These analyses were demonstrated using SPSS (v23; IBM, Japan) and the critical p-value for statistical significance was set at < 0.05.

Moreover, power analysis was performed to confirm whether the correlation between ΔMME and cumulative KAM was appropriately detected, with the statistical power calculated as 86%.

## Results

### Activity levels during jogging and daily activities on the time course

The average jogging duration was 36.3 ± 5.7 min. Moreover, the steps, pitch, and distances covered were 5985 ± 675.8 steps, 151.4 ± 11.9 steps / min, and 5.2 ± 0.15 km. On the other hand, during the pre- and post-effort periods, the average duration of daily activity was 1296 ± 169.7 min and 1328 ± 236.1 min, respectively. The average steps were 5719 ± 2629 and 7061 ± 3937, respectively (Table 2).

**Table 2:** Activity data classified by the established intervals

	Pre-effort	Effort	Post-effort
Time (min)	1296 ± 169.7	36.3 ± 5.7	1328 ± 236.1
Steps (n)	5719 ± 2629	5985 ± 675.8	7061 ± 3937
Length (km)		5.2 ± 0.2	
Pitch (steps/min)		151.4 ± 11.9	

Interval-pre-effort: T0 and T1, Interval-effort: T1 and T2, Interval-post-effort: T2 and T3. Pitch shows the average during effort. The values represent the mean ± standard deviation

### Reliability of ultrasound images

The ICC (1,3) between T0 and T1 in terms of supine MME and standing MME was 0.92 and 0.97, respectively. The cartilage thickness was 0.806 (*p* = 0.01).

### Effect of load stress on medial meniscus extrusion and cartilage thickness

**Fig. 4:**
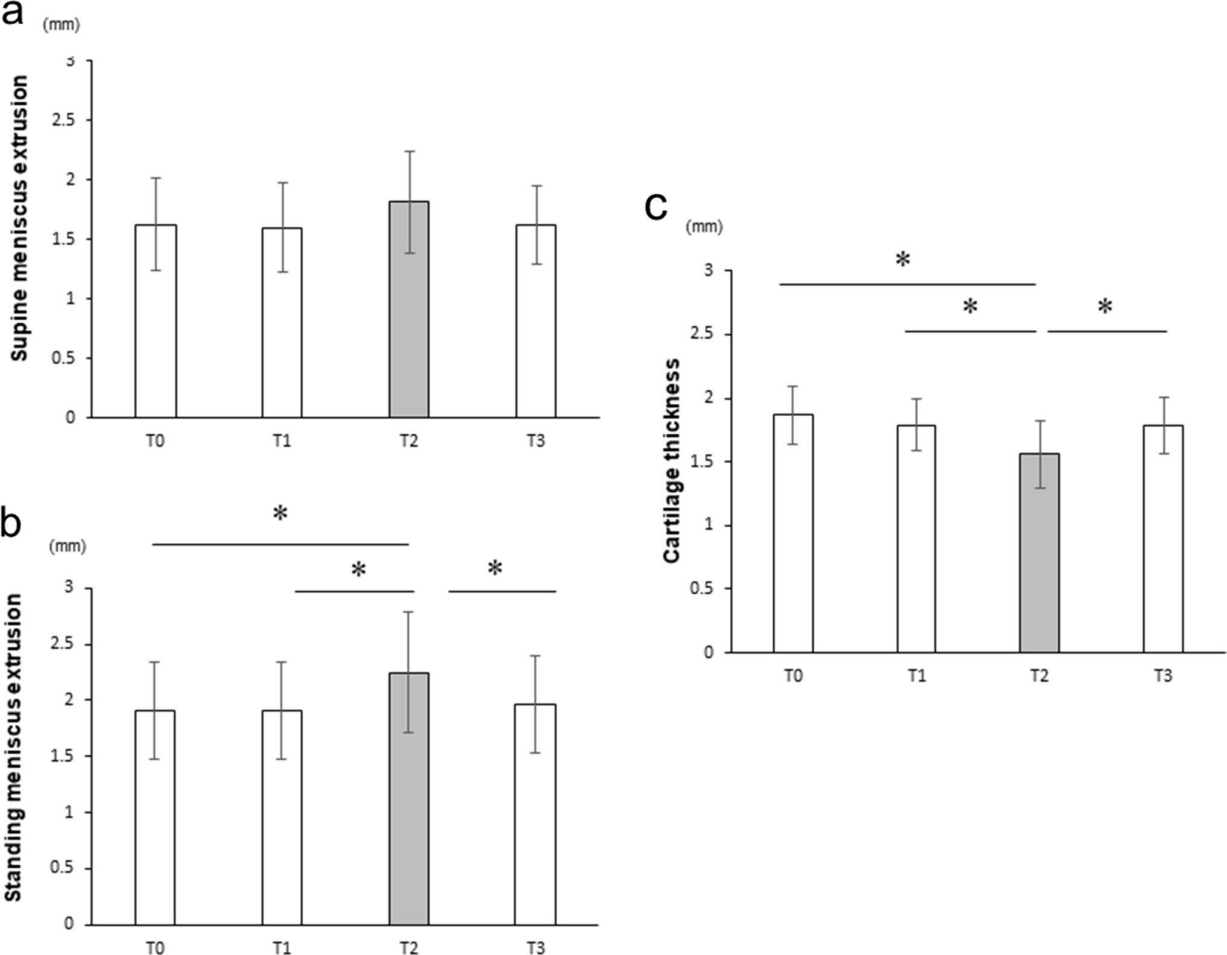
Meniscus and cartilage on the time course. The time course of MME in the supine (**a**) and standing (**b**) positions, and the cartilage thickness (**c**). These values show the mean ± standard deviation. * indicates a significant difference between conditions (*p* < 0.05)

There was no significant difference in MME between conditions in the supine position, whereas MME in T2 tended to be greater than those in T0 and T1 (Fig. 4a).

The MME in the standing position in T2 was significantly greater than those in T0 and T1. On the other hand, MME in T3 was significantly less than that in T2, and there was no significant difference with those in T0 and T1 (Fig. 4b). The cartilage in T2 was significantly thinner than that in T0 and T1. The cartilage in T3 was significantly greater than that in T2, and no significant difference was observed between those in T0 and T1 (Fig. 4c). The ΔMME was 0.2 ± 0.3 and 0.3 ± 0.2 mm in the supine and standing positions, respectively (Table 3). Moreover, the Δcartilage was -0.2 ± 0.2 mm.

**Table 3:** MME in each position and ΔMME

	T0	T1	T2	T3	Δ (T2-T1)
Supine MME	1.6 ± 0.4	1.6 ± 0.4	1.8 ± 0.4	1.6 ± 0.3	0.2 ± 0.3
*p*-value		1.0	0.277	0.408	
Standing MME	1.9 ± 0.4	1.9 ± 0.4	2.2 ± 0.5	2.0 ± 0.4	0.3 ± 0.2
*p*-value		1.0	0.02	0.017	

(T0): baseline, (T1) and (T2): before and after jogging uphill and downhill, (T3): 1 day after jogging, ΔMME: difference in MME between time points T1 and T2. The values represent the mean ± standard deviation

The Δcartilage was significantly correlated with the ΔMME in the supine and standing positions (supine *r* = − 0.56, *p* = 0.046; standing r = -0.69, p = 0.007) (Table 4).

**Table 4:** Correlation of evaluated parameters with ΔMMEs

	Supine ΔMME		Standing ΔMME	
	*r*	*p*	*r*	*p*
ΔCartilage (mm)	− 0.56	0.046	− 0.69	0.007
Peak KAM (Nm/kg)	0.15	0.62	0.43	0.139
KAM impales (Nms/kg)	0.2	0.52	0.52	0.069
Cumulative KAM (kNms)	0.38	0.196	0.68	0.01

*ΔMME* The difference in meniscus extrusion between before and after effort, *ΔCartilage* The difference in cartilage thickness between before and after effort, *KAM* knee adduction moment

*r* value shows the correlation coefficient with ΔMME

### Biomechanical data in the laboratory

In terms of laboratory-based measures, the speed and cadence were 1.7 ± 0.2 m/s and 159.5 ± 16.4 step/min, respectively. The peak and impulse of KAM during jogging were 0.8 ± 0.5 Nm/kg and 0.096 ± 0.07 Nms/kg, respectively. The cumulative KAM was 17.8 ± 11.8 kNms.

### Correlation between change of meniscus extrusion, cartilage, and cumulative stress

The cumulative KAM had a significant correlation with ΔMME in the standing position (*r* = 0.68, *p* = 0.01), but not for cartilage (*r* = − 0.44, *p* = 0.125). Moreover, there was no significant correlation between the peak KAM and ΔMME in the standing position (*r* = 0.43, *p* = 0.139) (Table 4).

## Discussion

The most important finding of the present study was that MME under standing conditions was greater at T2 compared with those at the other time points. Moreover, the greater MME after jogging (ΔMME) correlates with the cumulative KAM, which supports our hypothesis. The KAM is a critical indicator of mechanical stress on the medial compartment according to several previous studies [7, 8], demonstrating the quantitative load stress during the single stance phase of the gait cycle. Moreover, in other studies, the KAM correlated with greater MME [20, 27]. Additionally, cumulative KAM is known to have a greater impact on the medial compartment [9, 28]. Maly et al. reported that the cumulative knee adductor load, including the effects of daily steps in the individual information, was a more distinguishable parameter between individuals with and without knee OA, especially compared to the peak KAM [9]. Similarly, in the present study, the ΔMME in the standing position correlated with the cumulative KAM, but not with the peak value itself. Therefore, these results and previous studies indicate that the reaction of the extruded meniscus under weight-bearing conditions reflects the concentrated and cumulative stress on the medial compartment.

The MME was recovered at T3 and did not differ with those at T0 and T1. In general, the MME presents based on the medial meniscus posterior root and meniscotibial ligament lesions, i.e., dysfunction of meniscal attachment [29–32]. MME denotes meniscus instability and is considered a poor outcome of conservative treatment owing to its irreversibility [33, 34]. Conversely, the meniscus transiently extrudes and is escalated by extreme loading stress, such as that encountered during ultramarathons, even in healthy volunteers [21]. This reversible reaction of the MME may explain the changes of the meniscal extracellular matrix. The proteoglycan of the extracellular matrix provides elasticity [35, 36], and the collagen skeleton stabilizes the meniscus structure [37]. However, extreme mechanical stress depletes the proteoglycan [38] and leads to simultaneous temporary compromise in the collagen fibers. Therefore, due to the changes in the extracellular matrix due to extreme mechanical stress, the meniscal attachment may temporarily be stretched and subsequently recover before and after jogging. In this study, the activity data at the post-effort interval did not confirm the event including the extreme loading stress, thus indicating that the mechanical stress at post-jogging was less than that experienced during jogging. The healthy meniscus can therefore adjust and recover if it does not exceed the constant stress it encounters during daily activities.

In the biomechanical study, the control of cumulative mechanical stress is important for the prevention of knee injuries, cartilage degeneration, and progression of knee OA [1, 6]. Athletes always experience greater mechanical stress on their knees in various situations. However, the calculated cumulative mechanical stress has been obtained in the laboratory setting only and has not been established in a real-world setting. Therefore, it is important to establish a method to easily detect and visualize their cumulative mechanical stress to prevent injuries. In this study, MME was detected according to the various cumulative loads on the medial knee compartment. Interestingly, the cartilage showed a similar trend with the effect of loading stress on MME, whereas cumulative stress itself exhibited no correlation with cartilage deformation. The deformation and recovery of cartilage are associated with the change of the extracellular matrix based on cumulative mechanical stress [39], like the meniscus. However, Adams et al. reported that early-stage knee OA often showed a meniscus extrusion response rather than cartilage loss, indicating that the loss of cartilage is secondary to meniscus dysfunction [15]. Thus, the meniscus has an interstitial role in protecting the cartilage, indicating that meniscal function provides different cumulative stress to cartilage. Therefore, the reaction of meniscal extrusion that reflects the destruction of shock absolution is an essential indicator to detect knee pathology from initial stages.

Ultrasonography is cost-effective, portable, and adaptable, and can be used for evaluations immediately after jogging. However, its accuracy often depends on the examiner’s skill, for which the reliability must be established. In this study, the measurements were taken in adherence with established methods with high reliability [17, 24], and high ICC was also obtained. These results further indicated that ultrasound evaluations could detect the accurate reaction of the meniscus and cartilage and may therefore be useful for preventing injuries related to cumulative mechanical stress.

This study had several limitations. First, the sample size was small; therefore, the cutoff value of abnormal cumulative loads causing MME could not be determined. However, the current study can be the first step for determining the cumulative KAM. Second, MME is a better reflection of mechanical stresses during dynamic motion [40], and static evaluation might have underestimated its detection. Third, we included only healthy volunteers, which cannot be satisfactorily extrapolated to athletes, who perform specific activities competitively. Fourth, knee alignment data associated with KAM were not obtained. Finally, the cumulative KAM was obtained in a laboratory, where all tests were performed on flat surfaces. These data were not directly matched to uphill and downhill jogging. Future studies with larger sample sizes and including MME during motion and real motion biomechanical data are required to confirm our findings in a real-world setting.

## Conclusion

The temporary occurrence of meniscus extrusion observed on ultrasound correlates with the magnitude of cumulative knee adductor load in healthy volunteers.

## Data Availability

The data that support the findings of this study are available on request from the corresponding author on reasonable request.
