# Correction: Cumulative knee adduction moment during jogging causes temporary medial meniscus extrusion in healthy volunteers

**DOI:** 10.1007/s10396-025-01539-y

**Published:** 2025-04-09

**Authors:** Yosuke Ishii, Takato Hashizume, Saeko Okamoto, Yoshitaka Iwamoto, Masakazu Ishikawa, Yuko Nakashima, Naofumi Hashiguchi, Kaoru Okada, Kazuya Takagi, Nobuo Adachi, Makoto Takahashi

**Affiliations:** 1 Department of Biomechanics, Graduate School of Biomedical and Health Sciences, Hiroshima University, Hiroshima, Japan; 2 Department of Orthopaedic Surgery, Faculty of Medicine, Kagawa University, Kagawa, Japan; 3 Department of Musculoskeletal Ultrasound in Medicine, Graduate School of Biomedical and Health Sciences, Hiroshima University, Hiroshima, Japan; 4 Department of Orthopedic Surgery, Graduate School of Biomedical and Health Sciences, Hiroshima University, Hiroshima, Japan; 5 Ultrasound Business Operations, Healthcare Business Headquarters, KONICA MINOLTA, INC, Tokyo, Japan

**Correction: Journal of Medical Ultrasonics (2023) 50:229–236** 10.1007/s10396-023-01288-w

The article “Cumulative knee adduction moment during jogging causes temporary medial meniscus extrusion in healthy volunteers”, written by Yosuke Ishii, Takato Hashizume, Saeko Okamoto, Yoshitaka Iwamoto, Masakazu Ishikawa, Yuko Nakashima, Naofumi Hashiguchi, Kaoru Okada, Kazuya Takagi, Nobuo Adachi and Makoto Takahashi, was originally published under exclusive license to The Author(s), under exclusive licence to The Japan Society of Ultrasonics in Medicine. As a result of the subsequent decision to publish the article under the open access model, the article’s copyright notice was changed on 19 March 2025 to © The Author(s) 2023 and the article is now distributed under a Creative Commons Attribution 4.0 International License, which permits use, sharing, adaptation, distribution and reproduction in any medium or format, as long as you give appropriate credit to the original author(s) and the source, provide a link to the Creative Commons licence, and indicate if changes were made. The images or other third party material in this article are included in the article’s Creative Commons licence, unless indicated otherwise in a credit line to the material. If material is not included in the article’s Creative Commons licence and your intended use is not permitted by statutory regulation or exceeds the permitted use, you will need to obtain permission directly from the copyright holder. To view a copy of this licence, visit http://creativecommons.org/licenses/by/4.0

The original article has been corrected.

